# Endothelial cell apoptosis in chronically obstructed and reperfused pulmonary artery

**DOI:** 10.1186/1465-9921-9-19

**Published:** 2008-02-12

**Authors:** Edouard Sage, Olaf Mercier, Frederic Van den Eyden, Marc de Perrot, Anne Marie Barlier-Mur, Philippe Dartevelle, Saadia Eddahibi, Philippe Herve, Elie Fadel

**Affiliations:** 1 UPRES EA2705, Laboratoire de Chirurgie Expérimentale, Hôpital Marie Lannelongue, Le Plessis Robinson, France; 2 INSERM U841, Hôpital H. Mondor, AP-HP, Créteil, France

## Abstract

**Background:**

Endothelial dysfunction is a major complication of pulmonary endarterectomy (PTE) that can lead to pulmonary edema and persistent pulmonary hypertension. We hypothesized that endothelial dysfunction is related to increased endothelial-cell (EC) death.

**Methods:**

In piglets, the left pulmonary artery (PA) was ligated to induce lung ischemia then reimplanted into the main PA to reperfuse the lung. Animals sacrificed 5 weeks after ligation (n = 5), 2 days after reperfusion (n = 5), or 5 weeks after reperfusion (n = 5) were compared to a sham-operated group (n = 5). PA vasoreactivity was studied and eNOS assayed. EC apoptosis was assessed by TUNEL in the proximal and distal PA and by caspase-3 activity assay in the proximal PA. Gene expression of pro-apoptotic factors (thrombospondin-1 (Thsp-1) and plasminogen activator inhibitor 1 (PAI-1)) and anti-apoptotic factors vascular endothelial growth factor (VEGF) and basic fibroblast growth factor (bFGF) was investigated by QRT-PCR.

**Results:**

Endothelium-dependent relaxation was altered 5 weeks after ligation (*p *= 0.04). The alterations were exacerbated 2 days after reperfusion (p = 0.002) but recovered within 5 weeks after reperfusion. EC apoptosis was increased 5 weeks after PA ligation (*p *= 0.02), increased further within 2 days after reperfusion (*p *< 0.0001), and returned to normal within 5 weeks after reperfusion. Whereas VEGF and bFGF expressions remained unchanged, TSP and PAI-1 expressions peaked 5 weeks after ligation (*p *= 0.001) and returned to normal within 2 days after reperfusion.

**Conclusion:**

Chronic lung ischemia induces over-expression of pro-apoptotic factors. Lung reperfusion is followed by a dramatic transient increase in EC death that may explain the development of endothelial dysfunction after PE. Anti-apoptotic agents may hold considerable potential for preventing postoperative complications.

## Background

Chronic thromboembolic pulmonary hypertension (CTEPH) is due to chronic obstruction of large pulmonary arteries by organized blood clots after one or more episodes of acute pulmonary embolus [1,2]. CTEPH, initially thought to be rare, is being increasingly diagnosed, probably because effective medical and surgical treatments have been developed driven by progress in diagnostic tools [3]. Pulmonary thromboendarterectomy (PTE) is the treatment of choice for patients with CTEPH, as it restores perfusion to previously occluded zones and normalizes pulmonary vascular resistance [2]. However, PTE is associated with two major complications, persistent pulmonary hypertension and acute pulmonary edema, both of which can be related to endothelial cell (EC) dysfunction [4-6].

ECs play a pivotal role in preserving vascular integrity and preventing thrombosis. Good EC function is essential to maintain vascular homeostasis in health and disease. Apoptosis is among the biological processes that regulates EC number. EC death has been documented after ischemia and reperfusion in several organs [7,8]. Acute lung ischemia and reperfusion induced apoptosis in over 30% of parenchymal lung cells in humans and animal models after lung transplantation [9,10]. Thus, increased apoptosis may contribute substantially to the development of many of the adverse events seen after PTE, most notably pulmonary edema and persistent pulmonary hypertension.

The balance between pro-apoptotic and anti-apoptotic factors determines the overall amount of apoptosis. Therefore, we investigated whether expression of genes for pro-apoptotic and anti-apoptotic factors was affected by chronic lung ischemia and reperfusion. We studied two pro-apoptotic factors, thrombospondin-1 (Thsp-1) and plasminogen activator inhibitor-1 (PAI-1), and two anti-apoptotic factors, vascular endothelial growth factor (VEGF) and basic fibroblast growth factor (bFGF). Our working hypothesis was that abnormal expression of these pro-apoptotic and anti-apoptotic factors during chronic lung ischemia and reperfusion was associated with increased EC apoptosis and endothelial dysfunction. To evaluate this hypothesis, we investigated whether chronic pulmonary artery (PA) obstruction followed by reperfusion in piglets altered EC function and pulmonary vascular reactivity and/or EC apoptosis. Should such alterations be documented, we planned to investigate their mechanism, most notably the balance of pro-apoptotic and anti-apoptotic gene expressions. Finally, we investigated the effects of pentoxifylline, a nonselective anti-apoptotic factor, on EC viability and function in our ischemia/reperfusion model.

## Methods

### Experimental design

#### Groups

We used 50 piglets with a mean weight of 21.8 ± 3.9 kg. All procedures were approved by our institutional animal care committee.

In the first part of the study, we assessed EC apoptosis and endothelial function during chronic ischemia-reperfusion of the left lung induced by left PA ligation and re-anastomosis, which were performed as previously described [5,6]. The second part of the study investigated whether pentoxifylline prevented EC apoptosis and improved endothelial function after reperfusion.

The piglets were randomly divided into four groups of 10 animals. Animals were killed 5 weeks after ligation of the left PA (ligated group), 2 days after re-anastomosis of the left PA previously ligated for 5 weeks (acute reperfusion group), 5 weeks after re-anastomosis of the left PA previously ligated for 5 weeks (chronic reperfusion group), or 5 weeks after left PA dissection without ligation (sham group).

#### Tissue preparation

After heparin administration, the animals were killed by exsanguination. The left lung was removed from each animal and the PA flushed with 500 ml of 0.9% normal saline solution. The proximal PA was harvested down to the sub-segmental division, and samples from the peripheral third of the lung parenchyma were examined. The proximal and distal ECs of the left PA were examined separately.

Baseline wet-lung weight was estimated by measuring the weight of the left lung at the end of the experiment. They were then dried in an oven at 60°C. Dry weights were obtained after the weights no longer changed on successive weighings (i.e., after about 30 d). The wet to dry-lung weight ratio was then determinated.

### Detection of apoptotic cells

#### TUNEL assay

Cells undergoing apoptosis were detected using the ApopTag^® ^Red *In Situ *Apoptosis Detection Kit (Qbiogene, Illkirch, France), as specified by the manufacturer. Briefly, paraffin-embedded sections were deparaffinized and pre-treated with proteinase K (20 μg/ml) for 15 minutes. Equilibration buffer was added directly onto the specimen, after which terminal deoxynucleotidyl transferase (TdT) enzyme in reaction buffer was added for 1 hour at 37°C. Sections were washed in working strength Stop/Wash buffer for 10 minutes. Pre-warmed working strength anti-digoxigenin conjugate (rhodamine) was added to the sections and incubated at room temperature for 30 minutes. The samples were washed with PBS and observed under a fluorescence microscope after Hoechst staining (Sigma, Saint-Quentin Fallavier, France). Then, ECs in the proximal and distal PA were counted in a blinded fashion by two investigators working independently from each other, and the proportion of ECs undergoing apoptosis was calculated. The mean of the two counts by the two investigators was taken.

#### Caspase 3 Activity

Activity of the enzyme caspase 3 was measured using a colorimetric assay kit (R&D Systems, Lille, France) according to the manufacturer's instructions.

### Endothelial cell function

#### Pulmonary artery reactivity

At the end of the study, intrapulmonary arterial segments were dissected out, and endothelial relaxation was investigated as described previously [11]. Acetylcholine hydrochloride, calcium ionophore A23187, and sodium nitroprusside were used for relaxation after precontraction to phenylephrine.

#### Lung eNOS Protein

Endothelial nitric oxide synthase (eNOS) protein was measured by Western blot in homogenized lung tissue as described previously [11].

### Gene expression analysis by real-time quantitative RTQ-PCR

We used real-time quantitative polymerase-chain-reaction technology (RTQ-PCR) to measure the expression of genes for vascular endothelial growth factor (VEGF), basic fibroblast growth factor (bFGF), thrombospondin-1 (Thps-1), and plasminogen activator inhibitor-1 (PAI-1). RNA was extracted using Trizol reagent (Gibco Life Technologies, Maryland, USA). RNA concentration and quality were determined by electrophoresis on agarose gel and spectrophotometry. Then, reverse transcription was performed using random hexamer primers and reverse transcriptase (Biotech Ltd, UK). PCR primers were designed using Primer Express Software (Applied Biosystems, Foster, CA). To avoid inappropriate amplification of residual genomic DNA, intron-spanning primers were selected and internal control 18S rRNA primers provided. For each sample, the amplification reaction was performed in duplicate using SyberGreen mix and specific primers. Signal detection and result analysis were achieved using ABI-Prism 7000 sequence detection software (Applied Biosystems, Foster, CA). Expression of the gene of interest was computed relative to expression of the internal standard mRNA, r18S, using the following formula: relative mRNA = 1/2^(Ctgene of interest-Ctr18S)^.

### Effect of pentoxifylline

To investigate whether an anti-apoptotic drug, pentoxifylline [12], prevented EC damage and dysfunction, we studied 10 additional piglets, whose left PA was ligated for 5 weeks then reperfused for 2 days. A pentoxifylline bolus (40 mg) was injected into the left PA immediately before reperfusion. The results in this group (pentoxifylline group) were compared to those in the acute reperfusion group and sham group.

### Statistical analysis

Data are expressed as mean ± SD. One-way analysis of variance and simple linear regression studies were done using the Statview software package version 5 (Abacus Concept, Berkeley, CA). Probability values less than 0.05 were considered significant.

## Results

### Ischemia/reperfusion induced endothelial-cell apoptosis

**Figure 1:**
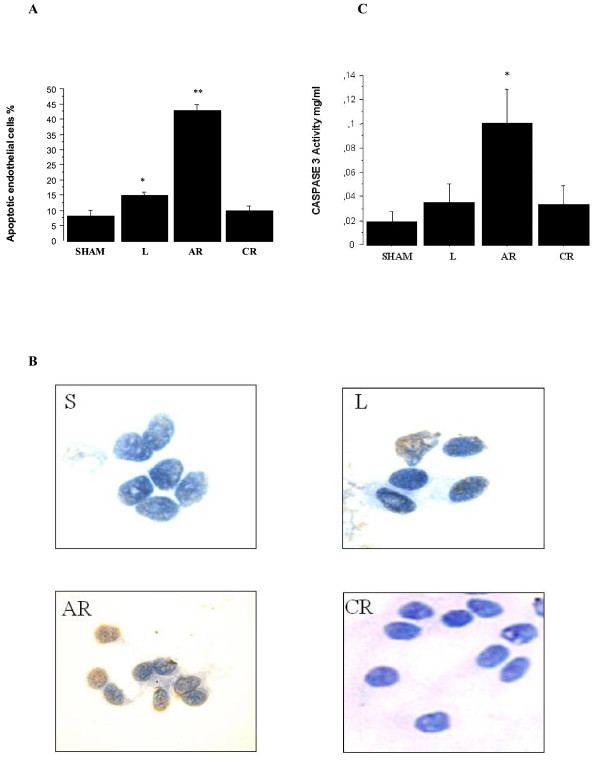
**A: **Proportion of endothelial cells undergoing apoptosis in the proximal pulmonary arteries. The proportion increased significantly from 8.1 ± 1.8% in sham animals (S) to 14.8 ± 1% 5 weeks after ligation of the left pulmonary artery (L), peaked at 42.9 ± 1.9% 2 days after reperfusion (AR), and returned to 9.8 ± 1.6% 5 weeks after reperfusion (CR). *p < 0.0001 versus sham, **p < 0.0001 versus all other groups. S: sham group; L: ligated group; AR: acute reperfusion group; CR: chronic reperfusion group. **B: **TUNEL staining of endothelial cells in the sham group, ligated group, acute reperfusion group after 2 days, and chronic reperfusion group after 5 weeks. Apoptotic cells are yellow. S: sham group; L: ligated group; AR: acute reperfusion group; CR: chronic reperfusion group. **C: **Caspase-3 activity in the proximal pulmonary arteries. Caspase-3 activity increased from 0.019 ± 0.008 mg/ml in sham animals (S) to 0.035 ± 0.015 mg/ml 5 weeks after ligation of the left pulmonary artery (L), peaked at 0.101 ± 0.028 mg/ml 2 days after reperfusion (AR), and returned to 0.034 ± 0.015 mg/ml after 5 weeks of reperfusion (CR). *p < 0.0001 versus sham.

Significant EC apoptosis was found in the proximal left PA 5 weeks after ligation (14.8 ± 1% versus 8.1 ± 1.8% in sham animals; *p *< 0.0001). The proportion of apoptotic endothelial cells peaked 2 days after reperfusion (42.9 ± 1.9%) and returned to control values 5 weeks after reperfusion (9.8 ± 1.6%) (Figure 1A, B)

Caspase-3 activity in the proximal PA followed a similar pattern, but the increase fell short of statistical significance at the end of the ischemic period (Figure 1C). Caspase-3 activity increased significantly after reperfusion (0.101 ± 0.028 mg/ml versus 0.019 ± 0.008 mg/ml in the sham animals;*p *< 0.0001) and returned to control values within 5 weeks after reperfusion (0.034 ± 0.015 mg/ml).

**Figure 2:**
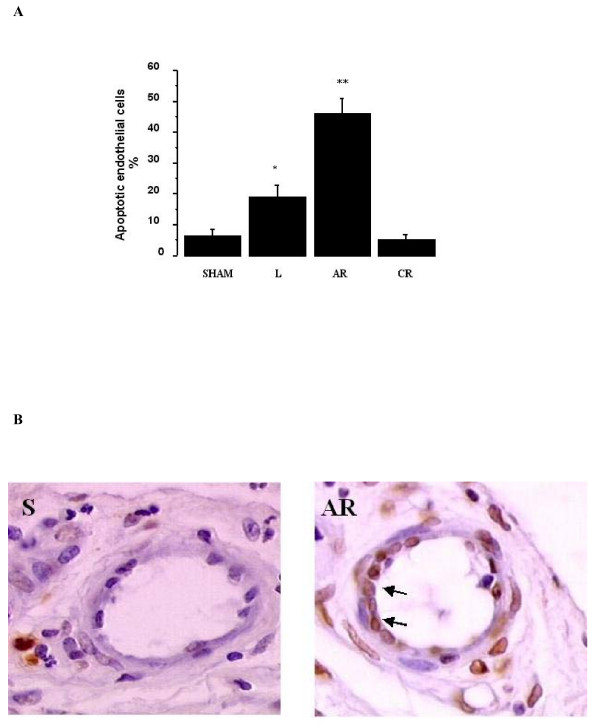
**A: **Proportion of endothelial cells undergoing apoptosis in the distal pulmonary arteries. The proportion increased significantly from 6.6 ± 1.8% in sham animals (S) to 18.9 ± 3.9% 5 weeks after ligation of the left pulmonary artery (L), peaked at 46.4 ± 4.5% 2 days after reperfusion (AR), and returned to 5.4 ± 1.5% 5 weeks after reperfusion (CR). *p < 0.0001 versus sham animals, **p < 0.0001 versus all other groups. S: sham group; L: ligated group; AR: acute reperfusion group; CR: chronic reperfusion group. **B: **TUNEL staining of endothelial cells in pulmonary artery branches ranging from 20 to 200 μm. Apoptotic cells are yellow (arrows). S: sham group; L: ligated group; AR: acute reperfusion group; CR: chronic reperfusion group.

Similarly, in the distal PAs, the proportion of apoptotic ECs increased significantly during the ischemic period (18.9 ± 3.9% versus 6.6 ± 1.8% in sham animals; *p *< 0.0001), peaked 2 days after reperfusion (46.4 ± 4.5%), and returned to control values within 5 weeks after reperfusion (Figure 2A, B). The proportions of apoptotic ECs in the proximal and distal PAs correlated significantly with each other (*p *< 0.0001, R = 0.97).

### Effect of ischemia/reperfusion on endothelial function

**Figure 3:**
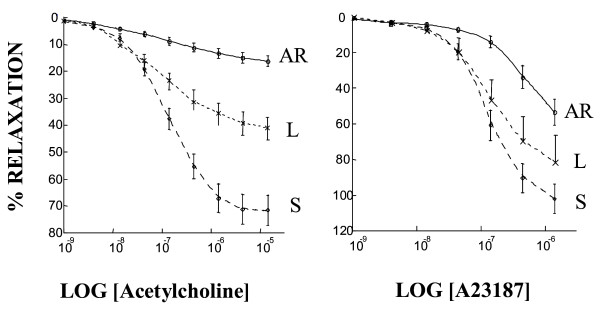
Relative eNOS protein expression as assessed by densitometric quantification of endothelial nitric oxide synthase on immunoblots prepared from lung preparations in the sham group (S) acute reperfusion group (AR).

**Figure 4:**
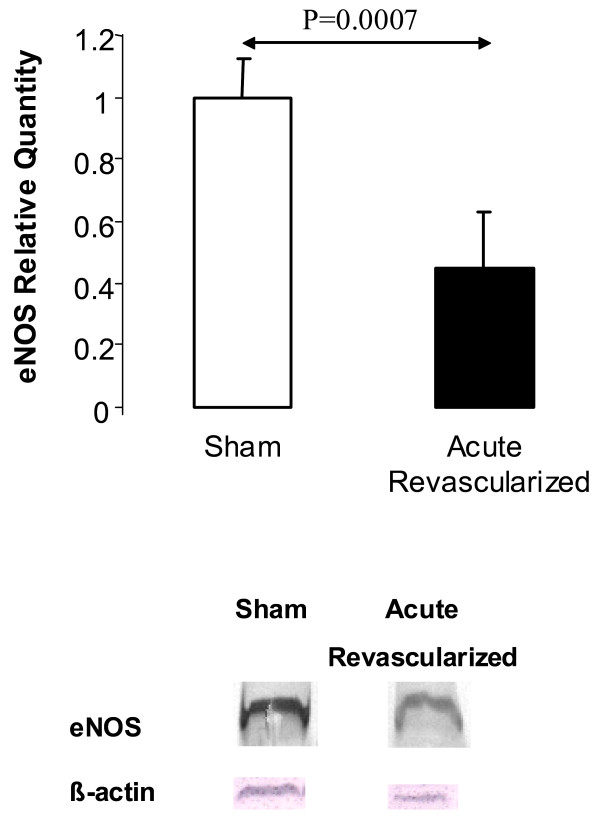
Percent reduction of maximal contraction to phenylephrine produced by acetylcholine stimulation and calcium ionophore stimulation of pulmonary artery rings taken from the left lungs of sham animals (S), animals with chronic ischemia (left pulmonary artery ligation for 5 weeks, L), and animals with acute reperfusion (revascularisation 2 days earlier, after ligation for 5 weeks, AR).

Neither PA contraction to phenylephrine nor PA relaxation response to sodium nitroprusside was affected by ligation or reperfusion. However, maximal relaxation in response to acetylcholine (Figure 3) was lower in the acute reperfusion group than in the sham (*p *= 0.0001) groups, although no significant differences in acetylcholine EC_50 _were noted across the three groups. Moreover, maximal relaxation in response to calcium ionophore (Figure 3) was lower in the acute reperfusion group than in the sham group. The EC_50 _to calcium ionophore was lower in the sham group than in the acute reperfusion group. The impairment in endothelium-dependent relaxation seemed correlated to the steady state of eNOS protein levels: eNOS protein was significantly decreased in the acute reperfusion group compared to the sham group (Figure 4).

Wet to dry ratios were not significantly different among the four groups.

### Gene expression in lung tissue from piglets with chronic ischemia/reperfusion

**Figure 5:**
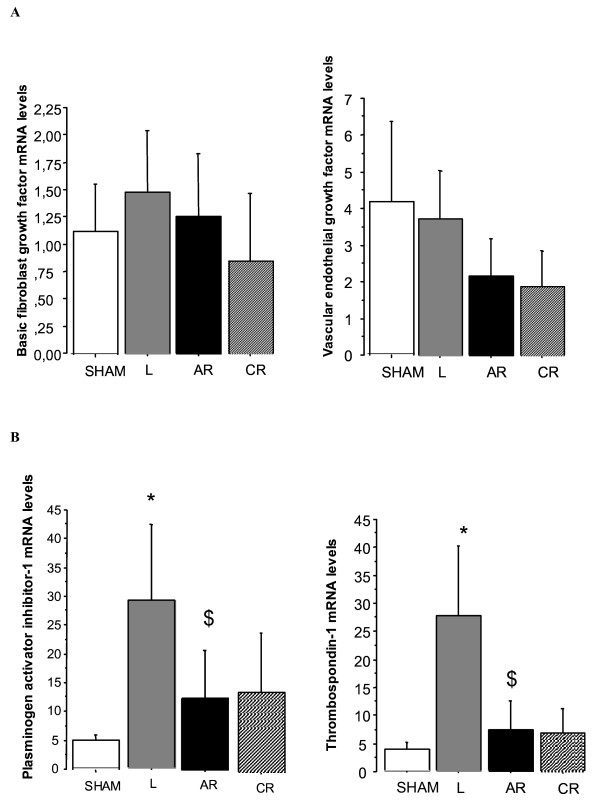
**A: **Relative expression of lung vascular endothelial growth factor and basic fibroblast growth factor mRNA in control piglets (sham) and animals exposed to chronic ligation (L) alone or followed by reperfusion for 2 days (acute reperfusion group, AR) or 5 weeks (chronic reperfusion group, CR). **B: **Relative expression of thrombospondin-1 and plasminogen activator inhibitor-1 mRNA in control piglets (sham) and animals exposed to chronic ligation (L) alone or followed by reperfusion for 2 days (acute reperfusion group, AR) or 5 weeks (chronic reperfusion group, CR). Expression levels were significantly increased 5 weeks after ligation of the left pulmonary artery (L) (*p = 0.0003 and *p = 0.0025, respectively) and significantly decreased after acute reperfusion (AR) ($*p *= 0.0008 and $*p *= 0.0163, respectively).

No changes in levels of VEGF or bFGF mRNA were detected during ischemia or reperfusion (Figure 5A). However, Thps-1 and PAI-1 mRNAs peaked at the end of the ischemic period (*p *= 0.0003 and *p *= 0.0025, respectively when compared to sham group). After reperfusion, expression levels decreased significantly (*p *= 0.0008 and *p *= 0.0163, respectively compared to the values found after the ischemic period) and returned towards normal values. (Figure 5B).

### Effect of pentoxifylline

**Figure 6:**
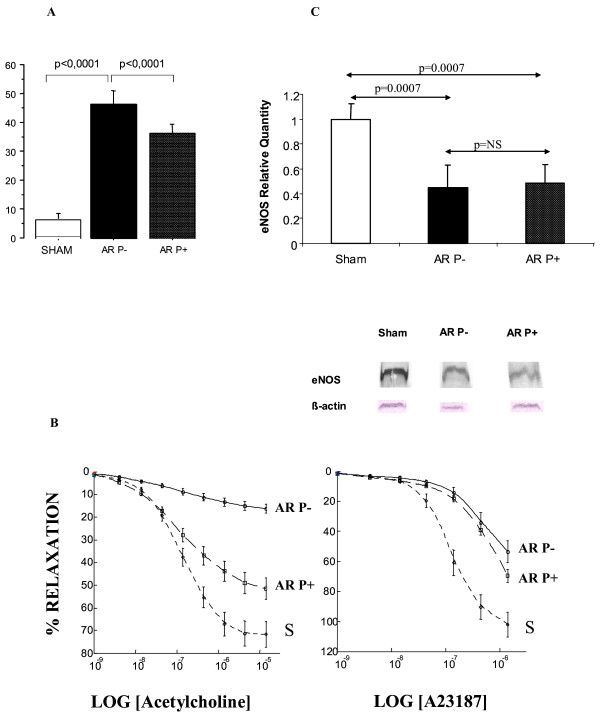
**A: **Proportion of endothelial cells undergoing apoptosis in the distal pulmonary arteries in the sham group and in piglets exposed to ligation for 5 weeks followed by reperfusion for two days, with (AR P+) or without (AR P-) pentoxifylline administration immediately before reperfusion. Typical results from two animals of each group are shown. **B: **Percent reduction of maximal contraction to phenylephrine produced by acetylcholine stimulation and calcium ionophore stimulation of pulmonary artery rings taken from left lungs 2 days after reperfusion with (AR P+) or without (AR P-) pentoxifylline administration (pentoxifylline+ and pentoxifylline-groups, respectively). Values are means ± SEM. The concentration-response curves were significantly flattened in the pentoxifylline-group and partially restored by administration of pentoxifylline. S: sham group; P-: acute reperfusion without pentoxifylline; P+: acute reperfusion with pentoxifylline. **C: **Western blot of eNOS protein content (mean ± SEM) 2 days after reperfusion with pentoxifylline (AR P+ group) or without pentoxifylline (AR P- group) compared to the sham group. Typical results from two animals of each group are shown.

Injecting pentoxifylline immediately before reperfusion resulted in a significant decrease in the proportion of apoptotic ECs in the distal PA, from 46.4 ± 4.5% to 36.3 ± 2.9% (*p *< 0.0001). However, the proportion of apoptotic cells remained higher than in the sham group (6.6 ± 1.8%; *p *< 0.0001) (Figure 6A). Neither contraction to phenylephrine nor relaxation to sodium nitroprusside were affected by PA ligation or reperfusion. However, maximal relaxation in response to acetylcholine (Figure 6B) was lower in the acute reperfusion group than in the pentoxifylline (*p *= 0.0001) and sham (*p *= 0.0001) groups; it was higher in the sham group than in the pentoxifylline group (*p *= 0.01). No differences in acetylcholine EC_50 _were seen across the three groups. Maximal relaxation in response to calcium ionophore was lower in the acute reperfusion group than in the pentoxifylline (*p *= 0.002) and sham (*p *= 0.0001) groups; it was higher in the sham group than in the pentoxifylline group (*p *= 0.001). The EC_50 _to calcium ionophore was lower in the sham group than in the acute reperfusion and pentoxifylline groups (1·35.10^-7 ^± 1.1·10^-7 ^M versus 3.39·10^-7 ^± 1.2·10^-7 ^M, *p *= 0.0001; and versus 4.07·10^-7 ^± 1.9·10^-7 ^M, *p *= 0.0001). The EC_50 _to calcium ionophore was similar in the acute reperfusion and pentoxifylline groups.

Reperfusion was followed by a significant decrease in eNOS protein (acute reperfusion group versus sham group). However, no significant difference was found between the acute reperfusion group and the pentoxifylline group (Figure 6C).

## Discussion

This study provides the first evidence that chronic lung ischemia followed by reperfusion is associated with significant EC apoptosis in proximal and distal PAs. EC apoptosis occurred during chronic lung ischemia, reached 46% 2 days after reperfusion, and returned to control values within 5 weeks after reperfusion. EC apoptosis was associated with significant endothelial function impairment, most notably regarding eNOS expression and nitric oxide synthesis. Our gene expression studies provided a likely explanation to the abnormalities in EC apoptosis and endothelial function. We found overexpression of the pro-apoptotic factors Thps-1 and PAI-1 after 5 weeks of PA ligation. Moreover, pentoxifylline injection before lung reperfusion protected against EC apoptosis and endothelial dysfunction. These data suggest prevention of EC death after reperfusion as a key target for treatments aimed at preventing the development of pulmonary edema and persistent pulmonary hypertension after PTE.

Apoptosis is the process of normal program to cell death that allows normal cell turnover and wall remodelling in blood vessels. Chronic PA occlusion, as occurs in CTEPH, may result in chronic pulmonary EC ischemia. However, the development of the bronchial circulation in areas of chronic PA occlusion has been shown to preserve pulmonary aerobic metabolism and to reduce the severity of lung oedema after reperfusion [6,11]. Interestingly, we showed that this systemic blood supply failed to prevent EC apoptosis in the reperfused PA bed, since almost half the ECs underwent apoptosis after reperfusion in our model. EC apoptosis occurred in both the proximal and the distal PA beds, as established not only by TUNEL assay but also by the dramatic increase in caspase-3 activation 2 days after reperfusion. This finding corroborates previous results made in the setting of acute ischemia-reperfusion injury of the lung and other organs [7-10].

Apoptosis is a quiet and well-organized cell death modality that is associated with less inflammation, compared to necrosis [13,14]. Apoptotic cells are phagocytized by macrophages before their membrane breaks down, so that their intracellular enzymes are not released. Apoptosis is triggered and modulated by two pathways. The intrinsic pathway involves the mitochondria and is activated by reactive oxygen species; whereas the extrinsic pathway is activated when ligands bind to their receptors, for instance tumour necrosis factor alpha (TNF-α) to TNF-receptors and Fas-ligand to Fas. Activation is rapid for the intrinsic pathway but may take up to several hours for the extrinsic pathway [14]. Increased apoptosis immediately after reperfusion in human lung transplantation was first reported by Fischer et al. in 2000, who found a time-dependent increase in apoptotic cell numbers after transplant reperfusion [9,10]. Although they found almost no positive TUNEL staining after cold or warm ischemia, the number of apoptotic cells increased over time after reperfusion. Subsequent studies established that cell death consistently peaked after reperfusion, both when acute ischemia was followed by transplantation and when chronic ischemia was followed by PA revascularisation. We found that nearly half the ECs were apoptotic 2 days after reperfusion and that control levels of apoptosis were recovered within 5 weeks. Similarly, in a rat lung transplant model over 30% of cells in the lung parenchyma were apoptotic 2 hours after reperfusion [10]. The peak in apoptotic cell number coincided with the peak of caspase-3 activity in our model, supporting a crucial role for the caspase pathway in cell death activation.

Increased EC death is the likely explanation to the endothelial function impairment, most notably the deficient nitric oxide production, documented in our study. A role for pro-apoptotic and anti-apoptotic proteins in pulmonary endothelial dysfunction has been hypothesized. This hypothesis, however, had not been previously studied in PA endothelium exposed to chronic ischemia. We found significant increases in mRNAs for two potent pro-apoptotic factors, Thsp-1 and PAI-1, in lung tissue exposed to chronic ischemia. In contrast, VEGF and bFGF remained unchanged. After reperfusion, pro-apoptotic protein expression returned to control values, although EC apoptosis increased to its peak. These results suggest that chronic lung ischemia induces overexpression of pro-apoptotic factors and that PA reperfusion after chronic ischemia may trigger massive EC apoptosis, leading to endothelial damage.

Our results indicating overexpression of pro-apoptotic proteins, including Thsp-1 and PAI-1, prompted us to investigate the effects of a nonselective inhibitor of EC apoptosis, pentoxifylline. Pentoxifylline is a potent anti-inflammatory agent that attenuates neutrophil-mediated lung injury and prevents EC dysfunction in several models of acute lung injury [12]. Pentoxifylline administration to patients with ischemic cardiomyopathy inhibited pro-inflammatory cytokines and reduced apoptosis by decreasing TNF-α and Fas concentrations in plasma [15]. Pentoxifylline probably prevents apoptosis both by inhibiting TNF-α production and by diminishing reactive oxygen species in our model. The extrinsic pathway can be activated during chronic ischemia via the release of cytokines (e.g., TNF-α) and of other proinflammatory inflammatory mediators in the lung, whereas the intrinsic pathway can be triggered after reperfusion by the release of reactive oxygen species.

Reperfusion pulmonary edema is a major cause of morbidity and mortality after PTE for CTEPH that requires in prolonged mechanical ventilation and may be fatal [16,17]. Increased permeability of the small lung vessels is the underlying mechanism. Onset is usually within 24 hours after reperfusion and is associated with neutrophil activation and sequestration in the lung [18]. Arterial hypoxemia and radiographic infiltrates in the reperfused pulmonary segments are noted. Severity is variable, ranging from mild reperfusion injury manifesting only as focal infiltrates to severe alveolar flooding. Treatment is primarily supportive, with mechanical ventilation and pharmacological support. Extracorporeal support has been used in selected patients with overwhelming reperfusion injury. Our results suggest that massive EC death shortly after reperfusion may cause pulmonary edema and pulmonary hypertension mediated by endothelial cell dysfunction [6,11]. Thus, EC apoptosis may be among the primary causes of reperfusion pulmonary edema and persistent pulmonary hypertension, the two main complications of PTE. Therefore, anti-apoptotic therapy holds promise for improving outcomes after PTE for CTEPH. Blocking the apoptotic cascade before reperfusion diminished ischemia-reperfusion lung injury and improved graft function [19]. Similar results were obtained with other organs, such as the heart and kidney [20,21]. Caspase inhibitors are now available commercially and their potential benefits in transplant patients are being evaluated in clinical trials.

In conclusion, our results show that chronic lung ischemia is associated with overexpression of pro-apoptotic genes. A 46% rate of apoptosis and significant endothelial dysfunction were noted 2 days after reperfusion of chronically ischemic PA endothelium. EC apoptosis was significantly reduced by pentoxifylline injected immediately before lung reperfusion, which significantly improved endothelial function. EC death may explain reperfusion pulmonary edema and persistent pulmonary hypertension occurring immediately after PTE. Therefore, anti-apoptotic therapy holds considerable promise for preventing the severe complications in patients undergoing PTE.
